# Model Membrane Systems Used to Study Plasma Membrane Lipid Asymmetry

**DOI:** 10.3390/sym13081356

**Published:** 2021-07-26

**Authors:** Haden L. Scott, Kristen B. Kennison, Thais A. Enoki, Milka Doktorova, Jacob J. Kinnun, Frederick A. Heberle, John Katsaras

**Affiliations:** 1 Large Scale Structures Group, Neutron Scattering Division, Oak Ridge National Laboratory, Oak Ridge, TN 37831, USA; 2 Shull-Wollan Center, Oak Ridge National Laboratory, Oak Ridge, TN 37831, USA; 3 Department of Chemistry, University of Tennessee, Knoxville, TN 37996, USA; 4 Department of Molecular Biology and Genetics, Cornell University, Ithaca, NY 14850, USA; 5 Department of Molecular Physiology and Biological Physics, University of Virginia School of Medicine, Charlottesville, VA 22903, USA; 6 Sample Environment Group, Neutron Scattering Division, Oak Ridge National Laboratory, Oak Ridge, TN 37831, USA; 7 Department of Physics and Astronomy, University of Tennessee, Knoxville, TN 37996, USA

**Keywords:** membrane asymmetry, cyclodextrin, molecular dynamics simulations

## Abstract

It is well known that the lipid distribution in the bilayer leaflets of mammalian plasma membranes (PMs) is not symmetric. Despite this, model membrane studies have largely relied on chemically symmetric model membranes for the study of lipid–lipid and lipid–protein interactions. This is primarily due to the difficulty in preparing stable, asymmetric model membranes that are amenable to biophysical studies. However, in the last 20 years, efforts have been made in producing more biologically faithful model membranes. Here, we review several recently developed experimental and computational techniques for the robust generation of asymmetric model membranes and highlight a new and particularly promising technique to study membrane asymmetry.

## 1. Introduction

The mammalian plasma membrane (PM) is composed of lipids and proteins, both peripheral and integral, that are chemically diverse and non-randomly distributed. Specifically, PM bilayer leaflets differ in the chemical compositions of their lipids [1]. For example, sphingomyelin (SM) and phosphatidylcholine (PC) are predominantly found in the PM’s extracellular leaflet, while phosphatidylserine (PS), phosphatidylethanolamine (PE), and phosphatidylinositol (PI) are primarily located in its cytoplasmic leaflet [1–4]. Importantly, the asymmetric distribution of charged PS and PI lipids generates an electrostatic potential that can influence protein-lipid interactions [5,6]. In addition to headgroup asymmetry, a marked asymmetry also exists within the acyl chain region. The extracellular leaflet is enriched in high-melting lipids possessing chains that are either fully saturated or contain a trans double bond, as in the case of the sphingosine moiety found in sphingolipids, including SM. In contrast, most phospholipids in the cytoplasmic leaflet possess one or more unsaturations in their sn-2 chains [7]. On average, this lipid asymmetry results in a PM with a more ordered extracellular leaflet, compared to its cytoplasmic counterpart [7,8]. As a result, the two lipid leaflets may have different physicochemical properties, which may be “communicated” across the bilayer midplane [9–11]. For example, it has been suggested that interleaflet coupling may induce lateral lipid rearrangement, altering the structure and dynamics of the membrane in ways that are very different from those of symmetric membranes [10,12,13].

Lipid asymmetry in the PM is actively maintained through different ATP-dependent transporters [14–17], such as flippases and floppases [14,18,19]. Since the cell expends much energy in actively inducing and maintaining lipid asymmetry, this PM feature is crucial for cellular viability and proper function [20,21]. An example of the biological importance of PM asymmetry is its induced loss by scramblases during cell apoptosis, a process that eventually leads to cell death [22]. Other examples involving the loss of PM lipid asymmetry but that are not related to apoptotic cell death are necrosis, ferroptosis, and necroptosis [23]. Interestingly, transient loss of PM lipid asymmetry has been reported in different cellular contexts, including intercellular communication, cell-cell contact, and intracellular signaling, further demonstrating membrane asymmetry’s vital role in a wide array of biological processes [24]. Finally, compromised membrane asymmetry is associated with a rare congenital bleeding disorder known as Scott’s Syndrome and is caused by a scramblase defect that impairs the transport of PS in platelet membranes, directly implicating the lipid composition and organization of the PM in health and disease [25].

Although the asymmetric distribution of lipids in the PM was discovered in the early 1970s [1,2], even today, practically all model membrane research has continued using symmetric membranes. The primary reason for this is the difficulty associated with preparing stable, asymmetric unilamellar vesicles suitable for biophysical studies. Although many biological insights have been obtained through studies of symmetric membranes, it is very likely that there are phenomena that only make themselves known when lipid asymmetry is present. Fortuitously, recent advances have made the generation of asymmetric model membranes much more practical, thus enabling one to study the intricacies of lipid-lipid and protein–lipid interactions under more biologically relevant conditions.

In the first part of this review, we will discuss recent techniques used to generate asymmetric model membranes *in vitro* that have advanced our understanding of the PM. In the second part, we focus on molecular dynamics (MD) simulations of asymmetric membranes.

## 2. Techniques Used to Prepare Asymmetric Membranes *In Vitro*

Since the discovery that the PM is asymmetric in its lipid composition [1], different methods have been used to create membrane asymmetry in model membranes. These include, but are not limited to, solid supported lipid bilayers (SLBs) [26], black lipid membranes (BLMs) [10], inverted emulsion vesicles (IEVs) [27], use of phospholipases or decarboxylases to modify lipid headgroups [28,29], ions to drive lipid redistribution [30,31], hemifusion between supported lipid bilayers and giant unilamellar vesicles (GUVs), resulting in asymmetric GUVs (aGUVs) [32], heavy-core small and large unilamellar vesicles (haSUVs and haLUVs), [33–35], and tensionless large unilamellar vesicles (aLUVs) [13,36–40]; the latter two methods use cyclodextrin-mediated lipid exchange to generate bilayer asymmetry. Not surprisingly, the asymmetric membranes produced by these different methods have their advantages and disadvantages (e.g., SLBs, BLMs, and IEVs suffer from potential artifacts arising from the presence of a support, residual oil, or both) [41]. However, of these methods, free-floating asymmetric vesicles offer the most biologically relevant platform to investigate the complex nature of PM lipid asymmetry, and its importance to biology [13,36–40].

### 2.1. Asymmetric Large Unilamellar Vesicles

#### 2.1.1. Ca^2+^ Ions Induce Asymmetry of PS Lipids in Membranes

**Figure 1.**
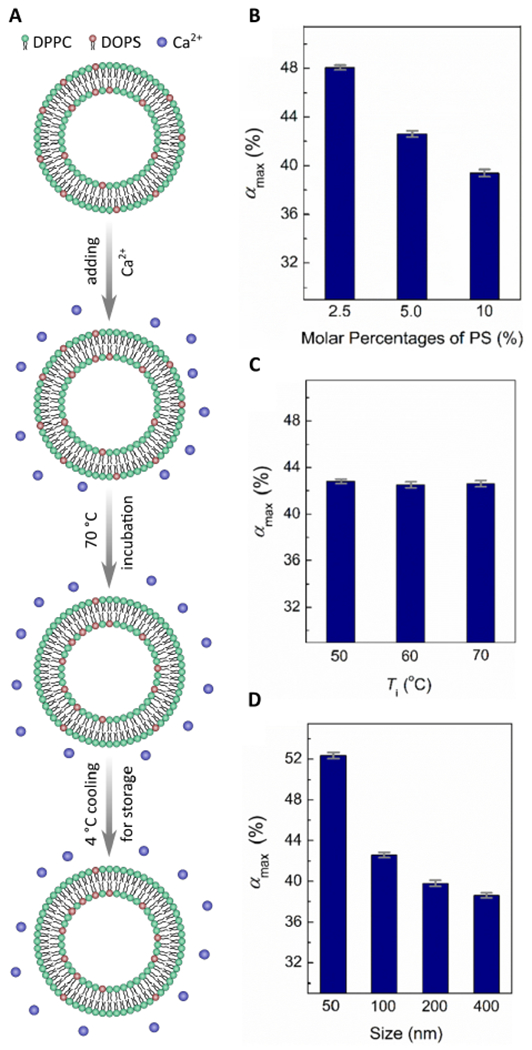
Ca^2+^ ions generate PS membrane asymmetry. (**A**) Scheme showing how Ca^2+^ is used to induce DOPS asymmetry in a DPPC/DOPS model membrane system. (**B–D**) The effect of mol% PS (**B**), temperature (**C**), and vesicle size (**D**) on asymmetry (labeled *α*_*max*_(%)). Smaller vesicles with less PS lead to a higher *α_max_*(%), whereas temperature does not seem to play a measurable role in lipid asymmetry. Figure adapted from [30,31].

Membrane asymmetry was first studied using SLBs or IEVs [10,26,27]. More recently, techniques producing model membranes that more faithfully mimic the PM have received much attention. For example, protocols have now been developed detailing the generation of freely floating asymmetric vesicles using Ca^2+^ ions to drive lipid redistribution (flip-flop) between the bilayer leaflets (Figure 1A) [30,31]. Specifically, 0.5 mM of Ca^2+^ was introduced to symmetric LUVs made from dipalmitoyl-PC (DPPC) and dioleoyl-PS (DOPS) lipids and incubated at 70 °C for about 40 h [30]. Under these conditions, PS lipids from the outer leaflet flip to the inner leaflet and remain there, thus increasing the concentration of PS in the inner leaflet vs the outer leaflet. This migration of PS from the outer to the inner leaflet is driven by Ca^2+^ ions bridging the headgroups of two PS lipid molecules, inducing a change in their intrinsic curvature. This newly formed PS-PS-Ca^2+^ complex favors the negatively curved inner bilayer leaflet over the positively curved outer leaflet. Using this sample preparation approach with a fluorescence quenching assay, Sun et al. studied the kinetics of lipid flip-flop and monitored both the extent and stability of bilayer asymmetry [30]. Ca^2+^ induced PS lipid asymmetry—with a ratio of PS*in*:PS*out* of 1.9—that was stable over a period of several days at room temperature. To further increase the PS*in*:PS*out* ratio, Ca^2+^ was only added to the extravesicular solution, resulting in a ratio of 2.3, an almost 20% increase [30].

A more detailed study by Guo et al. varied the lipid content, temperature, and vesicle size, further exploring the parameter space of Ca^2+^ induced PS asymmetry [31]. Guo et al. varied the starting mol% of PS lipids in symmetric vesicles and found that with increasing mol% PS lipids, *α* decreased, suggesting that increased amounts of PS do not translate to more asymmetry (Figure 1B). In contrast, temperature had practically no effect on asymmetry and *α* remained relatively constant as the incubation temperature decreased from 70 °C to 50 °C (Figure 1C). Finally, by adjusting the size of the vesicles, the degree of membrane asymmetry decreased with increasing vesicle diameter (Figure 1D) [31]. Although a promising technique to produce membrane asymmetry in model systems, this method has its limitations. Only negatively charged lipids, such as PS can flip from one side of the bilayer to the other via the electrostatic interactions induced between Ca^2+^ ions and their headgroups.

#### 2.1.2. Enzymes as Tools to Generate Asymmetric Vesicles

**Figure 2.**
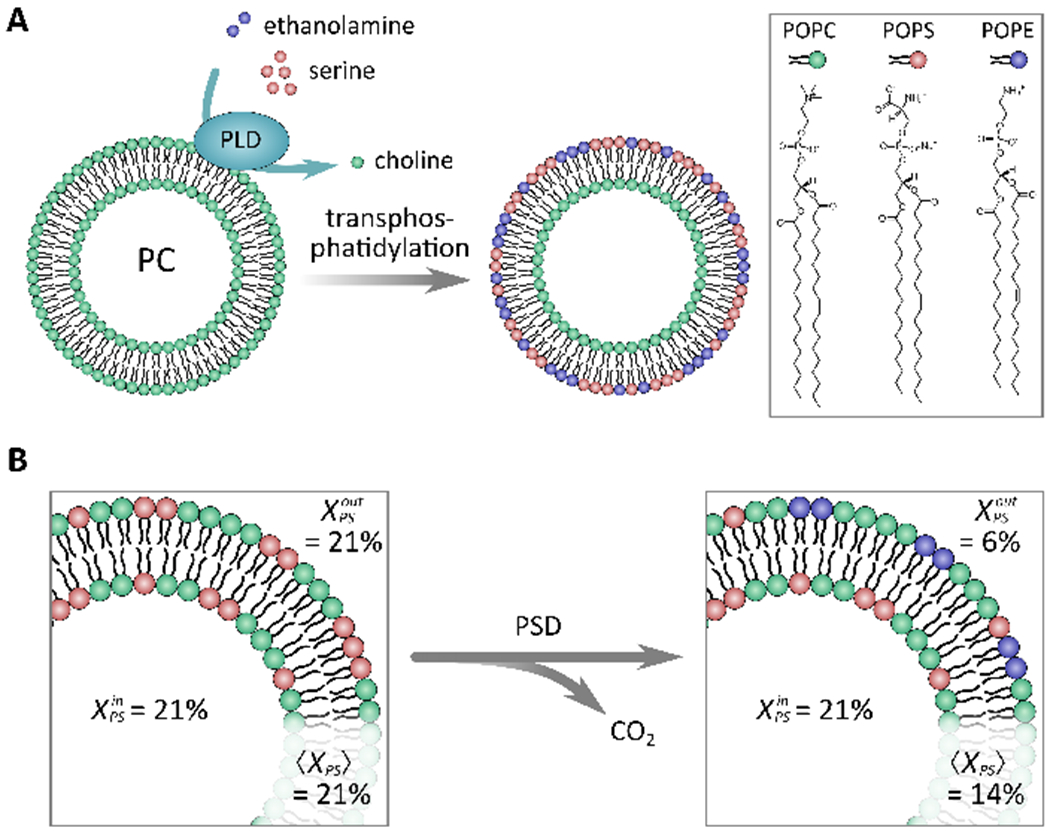
Enzymatically produced asymmetric model membranes. (**A**) Schematic showing the use of phospholipase D (PLD) and PC vesicles to prepare asymmetric model membranes rich in PS and PE in their outer leaflets, with PC populating their inner leaflets. (**B**) Illustration of the decarboxylation reaction of phosphatidylserine decarboxylase (PSD) used to convert a PS head group into a PE headgroup. When using PSD, most outer leaflet PS is converted into PE, producing a PS differential between the inner and outer leaflet of 14% PS. Adapted from [28,29].

There are different types of enzymes that can modify lipid headgroups to produce asymmetric membranes [28,29]. Takaoka et al. used phospholipase D (PLD) to convert PC lipids into ones with PS or PE headgroups via transphosphatidylation. To achieve this, PLD, serine, and ethanolamine were added to a solution of symmetric PC LUVs (Figure 2A) [29]. It was found that the amounts of PC converted to PS or PE by PLD were comparable regardless of incubation time (five hours vs. 24 h) [29]. Moreover, the addition of cholesterol (up to 30 mol%) did not largely affect headgroup conversion; however, cholesterol’s presence did increase the stability of the generated asymmetry [29]. In summary, using the described method, Takaoka et al. created an asymmetric membrane rich in PS and PE in its outer leaflet (inverse to what is found in the PM) that also includes cholesterol, a key membrane biomolecule [29].

Taking a similar approach used by Takaoka et al. [29], Drechsler et al. used phosphatidylserine decarboxylase (PSD) to convert PS lipids in the outer leaflet of symmetric PC/PS LUVs into PE (Figure 2B) [28]. Using this enzyme, Drechsler et al. were able to create vesicles with PS primarily in the inner leaflet and PE exclusively in the outer leaflet. PS asymmetry was quantified using *ζ*-potential measurements, as the loss of PS in the outer bilayer leaflet leads to a change in membrane potential [28]. They also observed that after ~80 min, the reaction reached an equilibrium and the addition of more PSD did not further alter the *ζ*-potential reading, suggesting that PSD had converted as much PS into PE as was possible. In the end, it was determined that 15 mol% of PS was converted into PE, with 6 mol% of PS remaining in the outer leaflet. Samples were found to be stable over days at 40 °C. As was demonstrated, using enzymes to generate asymmetric vesicles is quite effective; however, it is limited by the number of substrate lipids that these types of enzymes can react with. Additionally, the generated lipid asymmetry is inverse of what is found in the PM, making certain studies difficult to pursue.

#### 2.1.3. Using Cyclodextrins to Generate Asymmetric Vesicles

Discovered in the late 1800s, cyclodextrins (CDs) are a family of water-soluble cyclic oligosaccharides with amphiphilic properties. As a result of their physicochemical properties, CDs have been used, for example, in the food industry as stabilizers to extend product shelf life [42] and in the personal care industries as solubilizers that deplete cholesterol and lipids [43]. CDs have also been suggested for use in therapeutic strategies to treat diseases, including Alzheimer’s [44]. In membrane systems, including monolayers, bilayers, and living cells, CDs have largely been used to extract cholesterol [45]. Recently, there has been increased interest in using CDs to act as carrier molecules, shuttling lipids between different membrane environments. In the context of membrane asymmetry, CDs are used to exchange lipids from one pool of vesicles to another [13,33,36].

**Figure 3.**
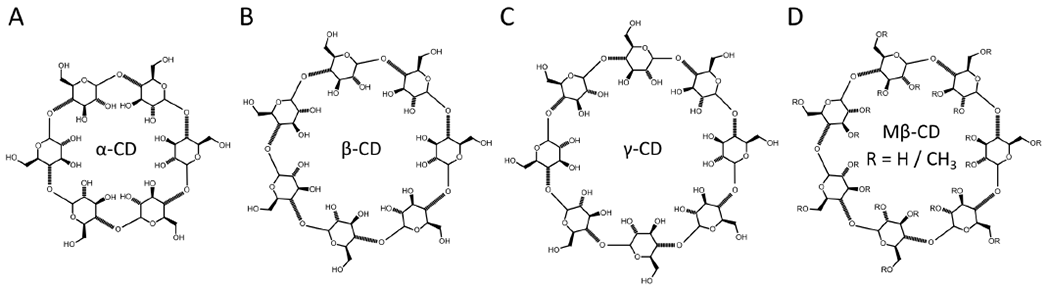
Chemical structures of different cyclodextrins (CD). (**A**) *α*-CD, (**B**) *β*-CD, (**C**) *γ*-CD, and (**D**) methyl-*β*-CD (M*β*CD). As the number of methyl groups in the ring increases, so does the size of the hydrophobic cavity.

CDs are composed of 6–8 oligosaccharides linked by 1–4 glycosidic bonds (*α*-, *β*-, and *γ*-CD, respectively). As a consequence of their cyclic structure, CDs have a hydrophilic exterior and a hydrophobic core. Binding of molecules takes place through the replacement of water with a hydrophobic molecule within the CD core, allowing for lipids to be removed from the membrane. The size of the CD core can vary, depending on the number of glucopyranose units, where the nomenclature *α*-, *β*-, and *γ*-CD refers to 6, 7, or 8 units, respectively, as shown in Figure 3. The variable core size enables some degree of selectivity with respect to the substrate molecule. For example, *α*-CD has a cavity that is slightly larger than the cross-sectional area of a lipid acyl chain, but smaller than the cross-sectional area of cholesterol, resulting in it having little to no affinity for cholesterol [35]. Alterations to CD structure can further influence selectivity, such as the randomly methylated CD variant, methyl-*β*-CD (M*β*CD) (Figure 3D) that has increased lipid affinity [43].

**Figure 4.**
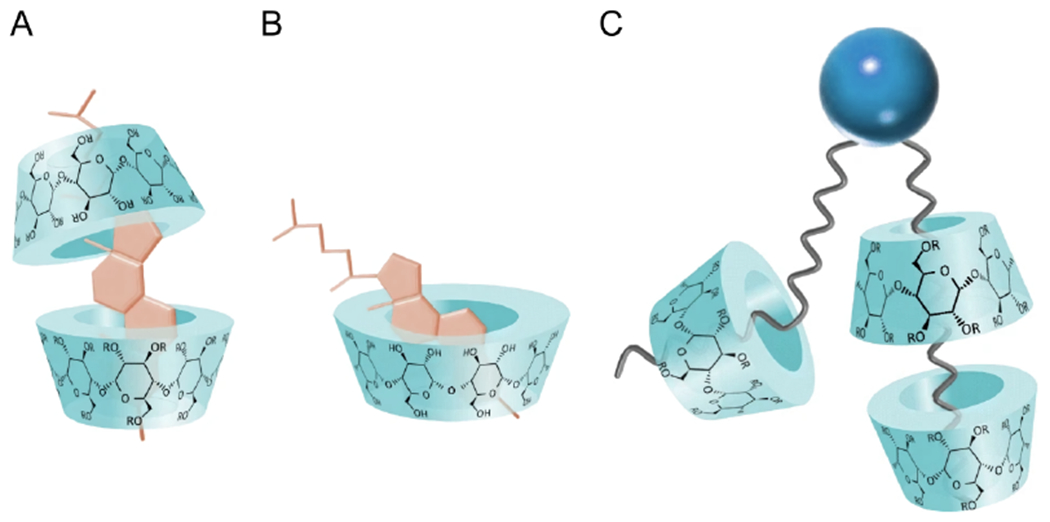
Schematic structures of different CD complexes. (**A**) 2:1 and (**B**) 1:1 Cd-cholesterol complexes. (**C**) A lipid-CD complex with more than one CD per lipid. In all cases, the CD hydrophobic pocket interacts with the hydrophobic regions of cholesterol and lipids. Adapted from [43].

What allows CDs to effectively interact with lipids are their hydrophobic cores that bind with the acyl chains of lipid molecules, allowing CDs to shuttle lipid molecules between donor and acceptor pools of vesicles (Figure 4). Using isothermal titration calorimetry (ITC), binding stoichiometry was determined showing the formation of lipid-CD complexes made up of more than one CD per lipid [46]. Additional studies have suggested that up to four CD molecules bind to one lipid molecule, implying that two CD molecules bind to each lipid acyl chain. However, this may be lipid specific [47] as lipids with unsaturated acyl chains have a lower affinity for CDs than their saturated counterparts–due to steric limitations introduced by double bonds (Figure 4C) [43]. Additionally, in the case of *α*-, *β*-, and *γ*-CD, studies have suggested the formation of cholesterol-CD complexes, where 1-2 CDs bind a single cholesterol molecule (Figure 4A,B) [48,49].

**Figure 5.**
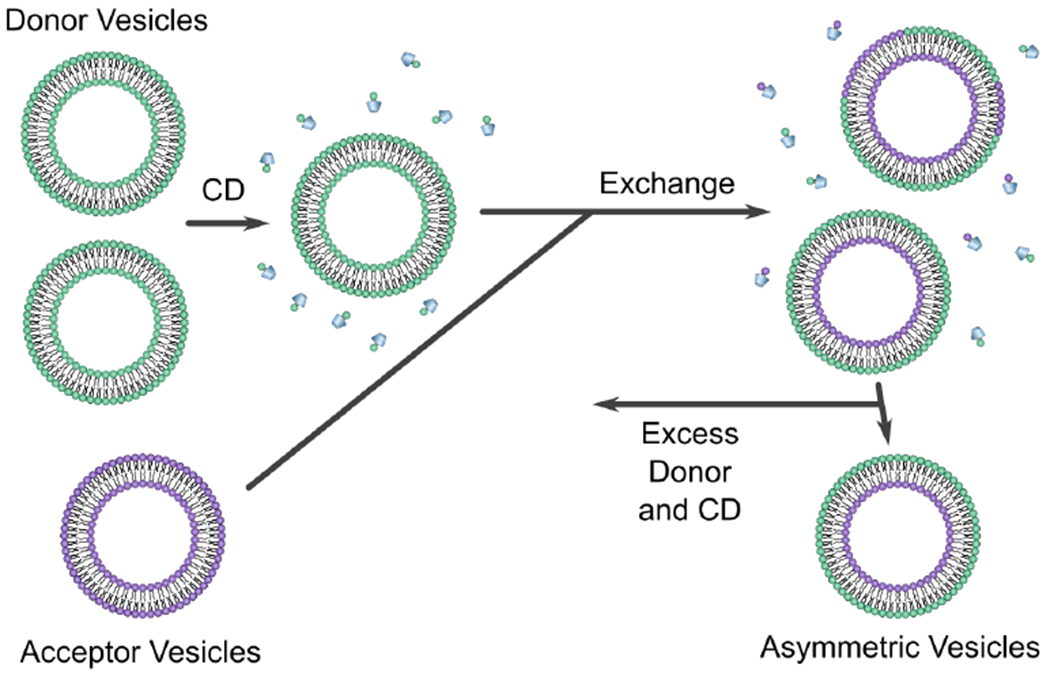
Schematic of the CD protocol used to prepare asymmetric vesicles. Donor vesicles with desired outer leaflet lipid composition are mixed with CD (represented by the trapezoids) and introduced to acceptor vesicles with the desired lipid composition in their inner bilayer leaflets. After incubating the Cd-lipid mixture, vesicles exchange their outer bilayer leaflets, resulting in asymmetric acceptor vesicles with the desired lipid compositions in both their inner and outer leaflets. Adapted from [50].

A particularly innovative protocol using CDs in model membrane research was developed by London’s group. In this method of asymmetric membrane preparation, symmetric acceptor vesicles with the desired inner leaflet lipid composition are introduced to an excess of symmetric vesicles with the desired outer leaflet lipid composition. In the presence of CD, the excess population “donates” lipids to the acceptor population. After sufficient time, the acceptor vesicles acquire outer leaflets with a lipid composition similar to the donor vesicles. The fraction of donor lipid in the outer leaflet of the acceptor vesicles is referred to as the exchange efficiency, which is dictated by experimental conditions and the relative affinity of CD for the donor and acceptor lipids. Following exchange, asymmetric vesicles are separated from the pool of donor vesicles and CD, as shown in Figure 5 [50].

Pioneering studies by the London group described the preparation of asymmetric heavy-core small unilamellar vesicles (haSUVs) with M*β*CD [33]. In this preparation, acceptor SUVs are produced with a sucrose core (haSUVs) to increase their density, thus facilitating the removal of the donor vesicles and CD by ultra-centrifugation. Fluorescence anisotropy measurements using TMA-DHP and DPH doped into haSUVs were carried out on haSUVs whose inner leaflet contained 1-palmitoyl-2-oleoyl PE and 1-palmitoyl-2-oleoyl PS (POPE:POPS) and SM or SM:cholesterol in their outer leaflet. These experiments revealed an asymmetric bilayer whose acyl chains were more ordered than the same lipid composition found in their symmetric counterparts (i.e., SM:POPE:POPS or SM:POPE:POPS:cholesterol). These results implied that SM or SM:cholesterol in the outer leaflet of haSUVs induce additional order in the inner leaflet (i.e., interleaflet coupling) [33]. Subsequently, London et al. extended their haSUV protocol to produce asymmetric large unilamellar vesicles or haLUVs [34,51,52].

haLUVs were used to study the coupling between bilayer leaflets in a system with liquid-disordered (Ld) domains of DOPC:cholesterol and liquid-ordered (Lo) domains made of high-melting temperature lipids (14:0 PC, 15:0 PC, 16:0 PC, and 18:0 PC) in their outer leaflets by monitoring their thermal stability; the inner bilayer leaflets of these haLUVs were composed of DOPC and cholesterol, mimicking the lipid environment of the PM. Interestingly, for the longest chain length PC (18:0 PC), the temperature at which the Lo domains melted was similar to the temperature measured in symmetric vesicles, suggesting that the inner DOPC:cholesterol leaflet does not, for the most part, affect the Ld domains. On the other hand, for haLUVs with 14:0 PC, the formation of Ld domains never took place, suggesting the presence of strong interleaflet coupling [52].

**Figure 6.**
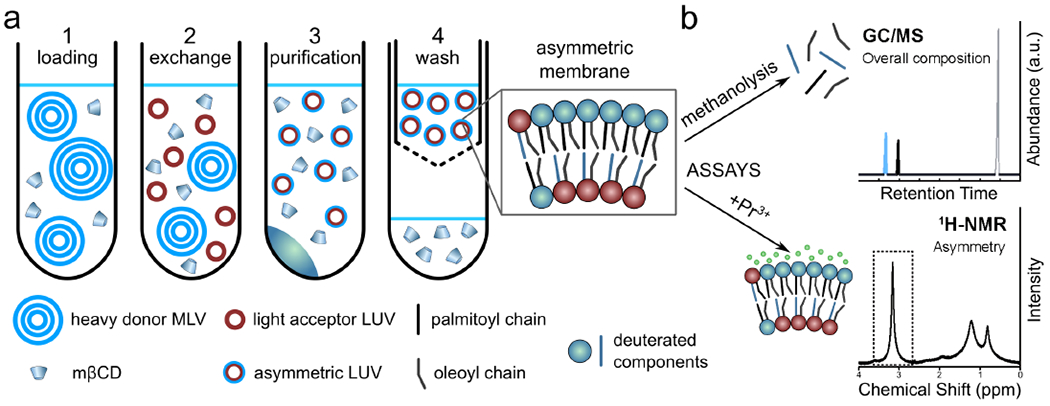
Schematic of the procedure used to prepare aLUVs and the analysis to determine. Panel (**a**): (1) Sucrose loaded MLVs are incubated with M*β*CD. (2) M*β*CD mediates the exchange of lipids between donor MLVs and acceptor LUVs. (3) Sucrose loaded MLVs are removed by centrifugation. (4) M*β*CD is removed using centrifugal concentrators, also allowing for buffer exchange. Panel (**b**): (Top) GC/MS is used to determine the overall membrane composition by quantifying the relative amounts of different acyl chains in the sample. (Bottom) ^1^H NMR in combination with Pr^3^+, a paramagnetic shift reagent that only interacts with the outer leaflet lipid headgroups, is used to quantify the degree of membrane asymmetry. Adapted from [13].

The haLUV protocol was later improved by removing the sucrose core of the acceptor vesicles, increasing the purity of the asymmetric liposomes, and implementing assays for the quantification of the individual leaflet compositions [13,36]. In contrast to the London et al. protocol, the donor vesicles were multilamellar vesicles (MLVs) with entrapped sucrose to increase their density (Figure 6(a1)). After incubation of donor and acceptor vesicles, low-speed centrifugation is used to sediment the heavy donor MLVs, as opposed to ultra-centrifugation used to pellet LUVs with a sucrose core (Figure 6(a2,3)). After the MLVs are discarded and CD is removed with centrifugal filtration, the aLUVs can be prepared in different buffers with different concentrations of deuterated water making them suitable for nuclear magnetic resonance (NMR) and small angle scattering experiments (Figure 6(a4)). Bilayer asymmetry and exchange efficiency are quantified using gas chromatography/mass spectrometry (GC/MS) and NMR spectroscopy (Figure 6b).

Applying this modified protocol to create aLUVs, Heberle et al. used small angle neutron scattering SANS and NMR to study the influence of a fluid inner POPC leaflet on a more ordered outer leaflet composed of POPC and DPPC [13]. GC/MS was used to quantify the exchange efficiency, as composition of the acyl chains, including length, unsaturation, and deuteration influence GC elution time. Additionally, the degree of asymmetry was determined using ^1^H NMR and the paramagnetic shift reagent Pr^3^+. When Pr^3^+ binds to the outer leaflet choline methyl groups, their resonances shift with respect to inner leaflet choline headgroups [37]. Of note is that Pr^3^+ does not cross the membrane on the timescale of the experiment and interacts only with the outer leaflet lipids [53]. Since the acceptor vesicles are composed of lipids with deuterated choline headgroups, the degree of asymmetry can be calculated from the fractional shift of the ^1^NMR choline resonance. This study provided two important findings. First, by comparing POPC vesicles prepared by conventional methods with those prepared by catalyzed exchange, it was shown that the protocol for generating bilayer asymmetry does not change membrane structure. Second, analysis of SANS data showed DPPC-rich domains in the outer leaflet were partially “fluidized” due to interleaflet coupling [13].

**Figure 7.**
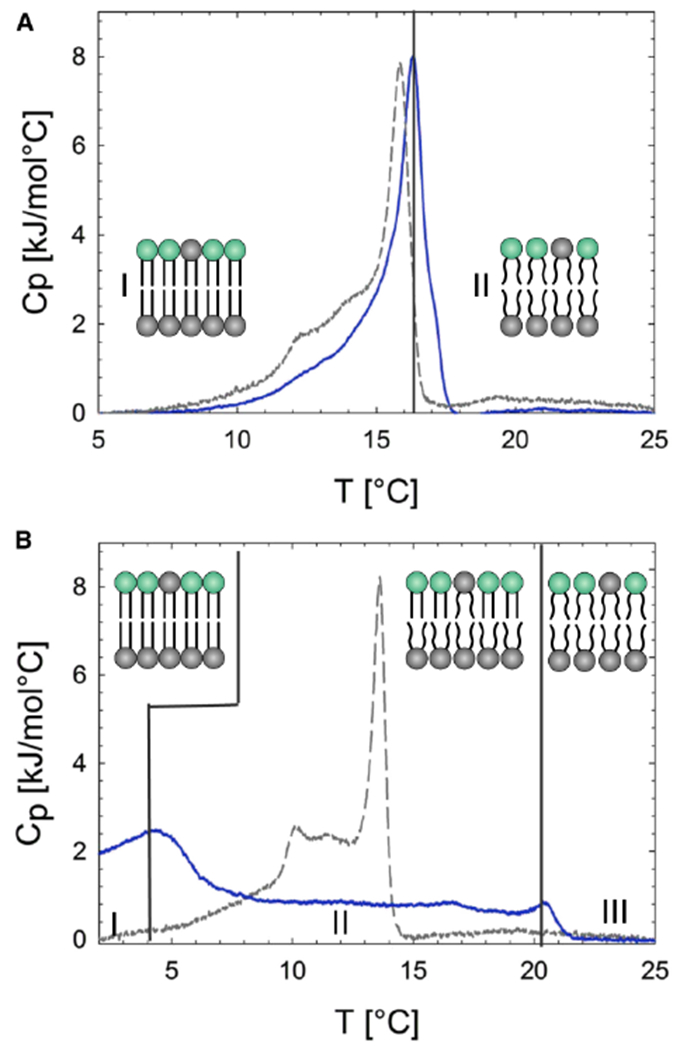
DSC cooling scans of aLUVs. Solid blue lines show DSC profiles of aLUVs prepared at a donor:acceptor ratio of 3:1. Dashed gray lines represent a scrambled lipid composition, where the aLUV lipids are mixed, resulting in symmetric LUVs. (**A**) DSC profiles of aLUVS with POPE in the inner leaflet and POPE:POPC in the outer leaflet. The regions (I) and (II) represents gel and fluid phase bilayers, respectively. Insets to the figure show schematics of gel and fluid phase bilayers. (**B**) DSC profiles of aLUVs with POPC in the inner leaflet and POPE:POPC in the outer leaflet. The DSC profile of aLUVs (solid blue line) can be described by three different regions. Region (I) represents gel phase bilayers. Region (II) corresponds to a co-existence of gel and fluid phase bilayers. Finally, in region (III), the bilayers are all in the fluid phase. Note that because lipid exchange efficiency is not 100%, the composition of the outer leaflet is a mixture of POPE:POPC. Adapted from [54].

Extending the study of aLUVs to more biologically relevant systems, Eicher et al. investigated the relationship between intrinsic lipid curvature and bilayer leaflet coupling [54]. For example, differential scanning calorimetry (DSC) scans were very different for aLUVs with POPE in the inner leaflet and POPE:POPC in the outer leaflet when compared to those where POPC populated the inner leaflet and POPE:POPC were found in the outer leaflet (Figure 7A,B). Interestingly, DSC scans of aLUVs with POPC in the inner leaflet showed two distinct transitions, suggesting the presence of uncoupled bilayer leaflets with coexisting gel and fluid phases (Figure 7B).

Recently, peptides have been incorporated into aLUVs to study peptide-membrane interactions. Looking first at the effect of membrane peptides on lipid flip-flop, Doktorova et al. [39] and Nguyen et al. [38] studied the loss of membrane asymmetry by incorporating gramicidin, alamethicin, melittin, or the pH low insertion peptide (pHLIP) into aLUVs. Using aLUVs composed of mostly DMPC in their outer leaflets and POPC in their inner leaflets and NMR, Doktorova et al. found that gramicidin increased the rate of lipid flip-flop by almost a factor of three [39]. Nguyen et al. extended this line of research by studying alamethicin, melittin, and pHLIP. Using the same aLUV composition as Doktorova et al., Nguyen et al. also found that the presence of gramicidin increased lipid flip-flop, but that membrane asymmetry was almost instantaneously destroyed in the presence of alamethicin, melittin, and pHLIP [38]. Taking a different approach, Scott et al. studied the effect that membrane asymmetry had on pHLIP— pHLIP is a pH sensitive peptide that inserts into the membrane, forming a transmembrane alpha helix at low pH [40,55]. Using aLUVs composed mostly of POPC in their outer leaflets and a mixture of POPC:POPS (mol ratio of 93:7) in their inner leaflets (similar composition to PM), Scott et al. found that the pH at which pHLIP inserts increased, where it was previously shown to decrease in the presence of POPS in symmetric vesicles, [40,55] suggesting that membrane asymmetry affects how membrane peptides and proteins interact with membranes.

**Figure 8.**
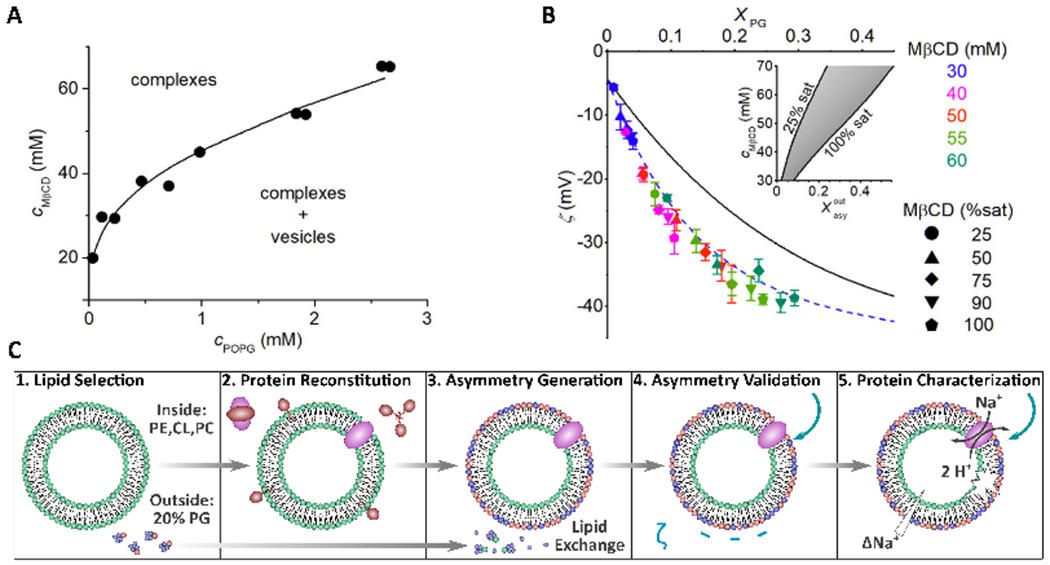
Using cyclodextrin-lipid complexes to accurately tune the composition of asymmetric vesicles. (**A**) Phase diagram of POPG-M*β*CD as determined by ITC. Black dots represent the phase boundary (saturation point) of various POPG to cyclodextrin ratios. (**B**) Using data from (**A**), M*β*CD concentration (M*β*CD (mM), POPG mol ratio in the exchange mixture (*χ_PG_*), and M*β*CD saturation (M*β*CD (%sat)) were varied to tune the desired amount of POPG in the outer leaflet of POPC vesicles, which was determined through *ζ*-potential measurements. Black and blue lines are of symmetric and asymmetric LUV calibration curves, respectively. As M*β*CD (mM), *χ_PG_*, and M*β*CD (%sat) are increased, either individually or simultaneously, the *ζ*-potential value becomes more negative consistent with a greater level of membrane asymmetry. Inset to the figure shows the range of M*β*CD concentrations needed to achieve the necessary mol% of POPG in the outer bilayer leaflets ($\chi_{\mathrm{out}}^{\mathrm{asy}}$) of POPC vesicles. (**C**) Schematic representation of the steps needed to form asymmetric proteoliposomes with a fully functional integrated protein. Adapted from [56,57].

Recently, the CD protocol was modified to better control the amount of donor lipid incorporated into the final asymmetric membrane. Previously, donor lipid in the form of MLVs was incubated with CD and the acceptor vesicles were directly added to this solution [13,33,36]. However, Markones et al. altered this protocol by instead treating acceptor POPC vesicles with donor lipid (1-palmitoyl-2-oleoyl PG (POPG))-cyclodextrin complexes in the absence of MLVs. Markones et al. first used ITC to determine POPG and cyclodextrin phase diagrams to ensure complete solubilization of the POPG vesicles (Figure 8A). Using the phase diagrams as guides, three parameters, CD concentration, donor lipid concentration, and CD saturation, were tuned to control the amount of exchangeable POPG in the system (Figure 8B). *ζ*-potential measurements were used to assay the extent and stability of POPG asymmetry, which was found to be stable for up to 14 days at 20 °C [56].

Markones et al. detailed a five-step protocol to prepare asymmetric proteoliposomes (Figure 8C). Specifically, they prepared asymmetric proteoliposomes with a lipid composition mimicking *Salmonella typhimurium* containing the Na+/H+ antiporter NhaA transmembrane protein that exhibited antiport activity in the presence of a substrate [57]. The work by Markones et al. now enables researchers to design asymmetric proteoliposomes and to compare the function of large transmembrane proteins in both symmetric and asymmetric membranes [56–58].

### 2.2. Giant Unilamellar Vesicles and Membrane Asymmetry

Giant unilamellar vesicles (GUVs) are between 10–50 microns in diameter, large enough to be imaged using optical microscopy techniques, allowing for the direct observation of bending fluctuations and membrane phase separation. Although symmetric GUVs can be produced with lipid compositions mimicking the composition of biological membranes, making asymmetric GUVs (aGUVs) poses a real challenge. The ability to prepare aGUVs, while a relatively new development, nevertheless holds great promise for revealing the biophysical consequences of an asymmetric lipid distribution. Continued aGUV innovation will undoubtedly open many doors for investigating the biological implications of asymmetry and interleaflet coupling.

#### 2.2.1. Preparation of aGUVs by M*β*CD-Mediated Lipid Exchange

To our knowledge, Chiantia and co-workers were the first to report on the preparation of aGUVs using M*β*CD-mediated lipid exchange [59], a method originally developed to produce haSUVs [33]. To prepare aGUVs with a DOPC inner leaflet and a brain sphingomyelin (bSM) outer leaflet, symmetric acceptor DOPC GUVs were prepared using electroformation [60]. A solution of donor lipid:CD complexes were prepared by incubating bSM MLVs with M*β*CD. The donor solution was then incubated with the acceptor DOPC GUVs, allowing donor lipids to exchange with acceptor lipids in the outer leaflets of the GUVs, resulting in aGUVs. Chiantia et al. used fluorescence correlation spectroscopy (FCS) to measure the diffusion coefficient of lipids in the inner and outer leaflets using the fluorescent probes NBD-DPPE and Atto647-acyl chain-labeled SM, respectively, thus informing about the nature of the lipid packing within each leaflet. After bSM was exchanged into the outer leaflet of symmetric DOPC GUVs, a decrease in lipid lateral diffusion was observed in the outer leaflet, but not in the inner leaflet, suggesting the presence of lipid asymmetry and a weak coupling between the leaflets. In contrast, when bSM was exchanged into the outer leaflet of brain phosphatidylcholine (bPC) vesicles, lateral diffusion decreased in both leaflets implying a stronger coupling between bilayer leaflets. Chiantia et al. suggested that the length of the acyl chains influences interdigitation and coupling between leaflets. For example, interleaflet coupling was more pronounced in milk-SM aGUVs, which have longer acyl chains (C22:0, C23:0, C24:0 -SM), compared to brain SM aGUVs (most abundant species is 18:0-SM). Incorporation of cholesterol into aGUVs via incubation with cholesterol-M*β*CD complexes resulted in a 40–50% decrease in lipid diffusion rates, comparable to the decrease observed in symmetric GUVs prepared from a 70:30 DOPC:cholesterol mixture [59].

#### 2.2.2. Cholesterol-Rich aGUVs Made Using M*β*CD-Mediated Lipid Exchange

Although cholesterol has a larger cross-sectional area than a single phospholipid acyl chain [61–63], the hydrophobic cavity of M*β*CD is large enough to facilitate the exchange of both cholesterol and phospholipids [64]. Controlling the composition of asymmetric vesicles composed of mixtures of these lipids is challenging when M*β*CD is used as the lipid carrier, due to its substantially greater affinity for cholesterol compared to phospholipids [65]. Nevertheless, using different GUV preparations Chiantia et al. were able to incorporate large amounts of cholesterol into aGUVs without destroying their asymmetry [59]. London et al. also used *α*-CD, which compared to M*β*CD has one less sugar group and a smaller hydrophobic cavity (Figure 3). Importantly, it only transports phospholipids and not cholesterol [45]. Hydroxypropyl-*α*-CD (HP*α*CD) was also shown to be effective in allowing efficient exchange between donor LUVs and cholesterol-containing acceptor LUVs without altering their cholesterol concentrations [51].

Interleaflet coupling using HP*α*CD was demonstrated using aGUVs containing 37 mol% cholesterol, with SM and DOPC in their outer leaflets, and DOPC in their inner leaflets. As a result of the Lo domains in the outer leaflet, there was formation of registered Lo domains enriched in cholesterol in the inner leaflet, a surprising result given that DOPC typically remains disordered when mixed with cholesterol in symmetric vesicles [66]. In the case of aGUVs with egg and milk-SM in their outer leaflets, NBD-DOPE was depleted in the Lo domains of both leaflets, implying that inner leaflet Lo domains were depleted in DOPC and enriched in cholesterol. For aGUVs containing egg-SM, Lo domains in the outer leaflet, but not the inner leaflet, were depleted in NBD-DPPE, suggesting that Lo domains in the two leaflets may differ in their physical properties. aGUVs that contained milk-SM in their outer leaflets had their Lo domains in both bilayer leaflets depleted in NBD-DPPE. Since milk-SM has long acyl chains that can interdigitate with the inner leaflet lipids, it was suggested that lipid interdigitation enhanced the coupling between lipids, resulting in lipid domains with similar physical characteristics [66].

#### 2.2.3. Phase Transfer, Water-Oil, Approaches Used in Generating aGUVs

**Figure 9.**
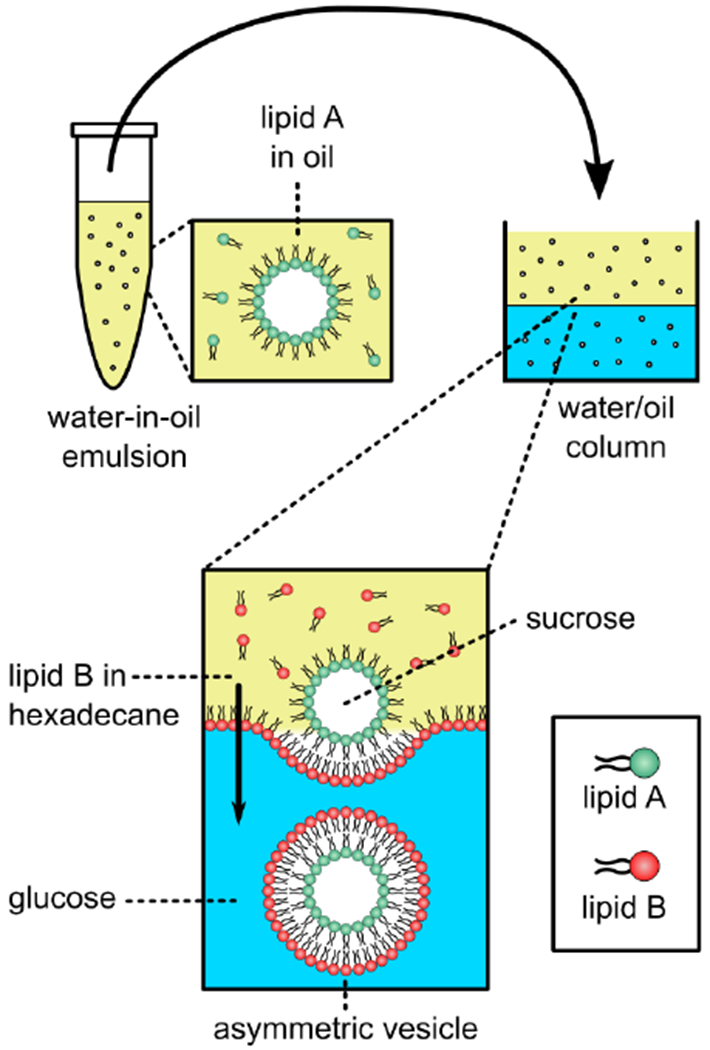
Schematic showing the water-oil approach to generate aGUVs. aGUVs are prepared in a two-step phase transfer procedure, in which water-in-oil droplets coated with one type of lipid are passed through a water-oil column with a lipid monolayer of a different composition, resulting in aGUVs. Adapted from [67].

In addition to CD-mediated lipid exchange, inverted emulsion phase transfer approaches that have been used successfully in synthetic biology have been used to generate aGUVs [67]. This work builds from inverted emulsion-based techniques for preparing asymmetric vesicles [27,68–70]. Symmetric GUVs can be produced in a two-step phase transfer procedure, in which water-in-oil droplets coated with lipid are passed through a water-oil column coated with a lipid monolayer at the interface, turning the droplets into GUVs (Figure 9). Changing the lipid composition in the oil phases produces aGUVs.

Using the phase transfer method, Elani et al. prepared POPC aGUVs with their inner and outer leaflets labeled with NBD-PE and Rh-PE (1,2-dipalmitoyl-sn-glycero-3-phosphoethanolamine-N-lissamine-rhodamine B sulfonyl) (ammonium salt), respectively [67]. Asymmetry was confirmed by fluorescence microscopy analysis of asymmetric hemifused GUVs that occasionally occur when two water-in-oil droplets pass through the oil/water interface together. In the hemifused structure, the middle bilayer separating the two halves of the vesicle contained only the inner leaflet NBD-PE fluorophore, indicating it was composed only of the inner leaflet lipid. In aGUVs with POPC outer leaflets and DOPC inner leaflets, or vice versa, Elani et al. observed that these aGUVs were much more rigid than symmetric GUVs of DOPC:POPC prepared either by electroformation or phase transfer. This surprising result was explained in terms of the lipids with different spontaneous curvatures and that asymmetry largely affects the membrane’s bending rigidity [67].

Similar results to Elani et al. were reported by Lu et al., where aGUVs with DOPC and DMPC in their inner and outer leaflets, respectively, were shown to have bending moduli that were about 50% greater than their symmetric counterparts [71]. According to the authors, this is because the curvature elastic energy is dependent on the bending modulus of each monolayer. Curvature elastic energy is smallest when the bilayer is compositionally symmetric. Deviations from this scenario cause the curvature elastic energy to increase, and therefore the bending modulus to increase [71].

An alternative and intriguing explanation proposed by Hossein and Deserno is that a differential stress in the asymmetric vesicles, possibly caused by an imbalance in the lipid packing density of the two leaflets, is responsible for the increased bilayer stiffness [72]. In either case, the experimental results show that asymmetry can have marked effects on membrane properties. Of note, is that the phase transfer approach for producing asymmetry may also introduce residual oil in the asymmetric vesicles. However, the fact that the bending rigidities between electroformed and phase transfer vesicles are practically the same indicates that residual oil is likely not affecting the mechanical properties of the vesicles.

#### 2.2.4. Hemifusion and aGUVs

A recent method for preparing aGUVs uses calcium-induced hemifusion of symmetric GUVs to a supported SLB [32]. This method of producing asymmetric membranes addresses several previous issues, namely it does not make use of possible contaminants, such as organic solvents and CDs. Notably, the method yields aGUVs with a broad range of membrane asymmetry.

**Figure 10.**
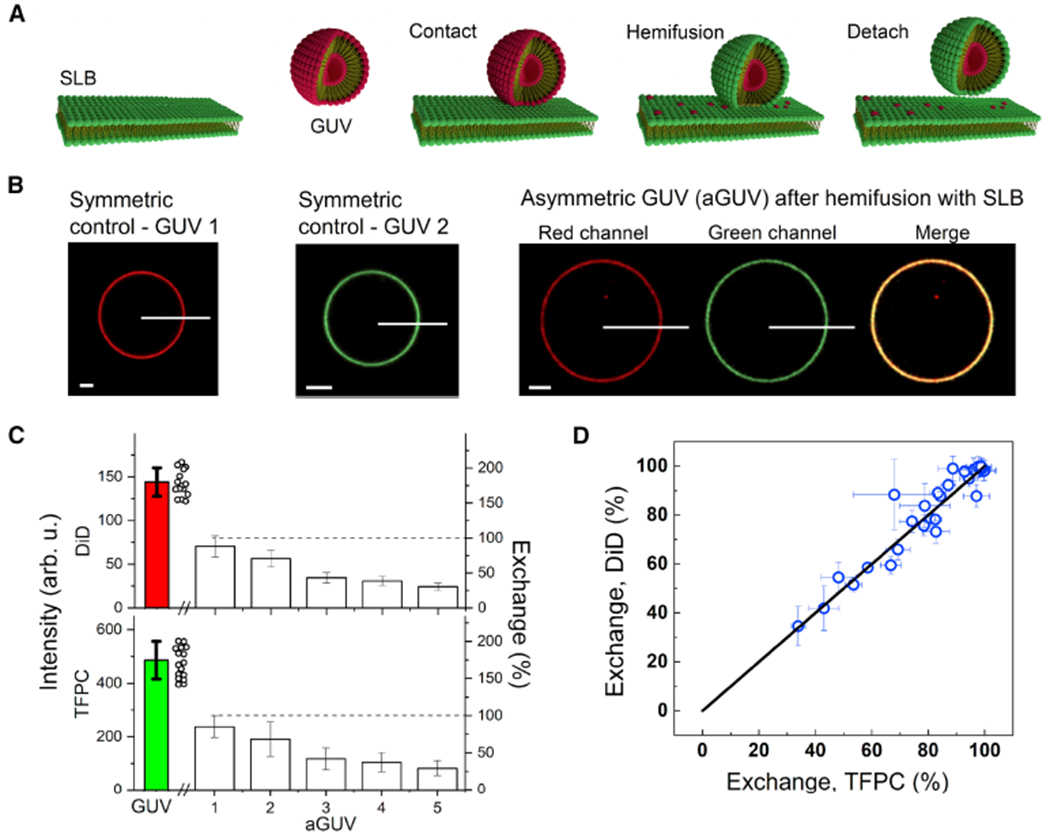
Schematic showing how hemifusion generates aGUVs. (**A**) Calcium induces hemifusion between a symmetric red-labeled GUV and a green-labeled SLB. (**B**) Comparison of a “control” symmetric GUV and an aGUV after hemifusion using the different fluorescence intensities. (**C**) Bar graphs representing the intensity of each fluorophore in symmetric GUVs (filled bars) and aGUVs (open bars). Open dots represent the different symmetric GUVs measured. (**D**) Percent exchange determined from each fluorophore showing lipid exchange. There is a range in the asymmetry of individual aGUVs that is accurately determined using this method of aGUV preparation. Adapted from [32].

To begin, symmetric GUVs are first prepared via electroformation [73] and a red fluorophore is added for visualization by confocal microscopy (Figure 10A). An SLB composed of different lipids plus a green fluorophore is prepared by vesicle fusion onto a chambered cover glass (Figure 10A). After a small aliquot of the GUV suspension is added to the SLB chamber, hemifusion is initiated through the addition of a calcium-containing buffer (Figure 10A). During this step, the distal leaflets of the hemifused GUV and SLB are joined, allowing for lipid exchange (the GUV’s inner leaflet remains unperturbed during this step) (Figure 10A). EDTA is added to chelate the calcium to stop further hemifusion from occurring, followed by the termination of hemifusion driven by gentle shearing of the now-asymmetric GUVs from the support for subsequent imaging (Figure 10A). The amount of lipid exchanged is determined by comparing intensities of each fluorophore (red and green) in the aGUV to those of symmetric GUVs containing only the red or green fluorophore, providing two independent measurements of the extent of bilayer asymmetry (Figure 10B). A dithionite quenching assay is used to determine if the lipids exchanged between the GUV and SLB were found in the external leaflet of the aGUV [32].

Enoki et al. examined aGUVs with an internal leaflet composed of DSPC:DOPC: cholesterol that phase separates into Ld and Lo phases, thus mimicking the exoplasmic leaflet of the PM. After exchange with a DOPC:cholesterol (80:20 mol%) SLB, the external leaflet of aGUVs was found enriched in the low-melting DOPC lipid and depleted in the high-melting DSPC lipid, as determined by changes in the intensities of the red and green fluorophores in the aGUVs after lipid exchange (Figure 10C). By plotting the percent exchange of each fluorophore, the level of asymmetry between the different aGUVs can be determined (Figure 10D). Interestingly, “induced ordered domains” were observed in all aGUVs, regardless of the extent of lipid exchange, suggesting that ordered domains were formed in the external DOPC-rich leaflets of aGUVs that would otherwise form a uniform Ld phase in symmetric bilayers. Enoki et al. also reported that “induced ordered domains” in aGUVs were less ordered than Lo domains in the initial symmetric phase-separated vesicles, suggesting that interleaflet coupling influences domain properties in asymmetric bilayers [32].

The factors responsible for the size of domains is a topic of great interest. In the case of symmetric vesicles, line tension plays an important role in controlling domain size [74]. For example, symmetric model membranes made of DSPC:DOPC:cholesterol form micron-sized domains. However, the partial replacement of DOPC by POPC leads to a decrease in line tension, as shown by flicker spectroscopy measurements [74]. Furthermore, replacing DOPC by POPC leads to the formation of nanodomains as reported by several different studies [75–77]. Interestingly, Heberle et al. observed a bilayer thickness mismatch between the Ld and Lo phases in the DSPC:DOPC:cholesterol system that decreased as a function of increasing POPC and decreasing DOPC [76]. For hemifused aGUVs with a DOPC-rich outer leaflet, the line tension was found to be less than that in their symmetric counterparts [78]. These data may give some indication as to how cells may control the existence and size of lipid rafts in the PM, namely by regulating the lipid composition in their bilayer leaflets.

## 3. Molecular Dynamics (MD) Simulations Used to Model Asymmetric Membranes

Despite recent technological advances in the preparation of asymmetric model membranes *in vitro,* the limited resolution of the experimental approaches leaves many questions pertaining to bilayer asymmetry, unanswered. In that respect, molecular dynamics (MD) simulations have emerged as an indispensable tool for complementing experiments and helping to fill existing knowledge gaps (see e.g., [39,79,80]). MD can be used to simulate the dynamics of both symmetric and asymmetric lipid bilayers under different conditions. The molecular models used by MD can either be fully atomistic or more coarse-grained, thus offering different levels of detail and the opportunity to examine various modes of membrane dynamics by simulating bilayers of different sizes and shapes [5,81–83].

Simulations of relatively flat bilayer patches have been most accessible and commonly used to study membrane asymmetry (see all studies referenced below). However, it is important to note that these simulations are normally conducted under periodic boundary conditions that mimic an infinite bilayer plane in the lateral direction and stacked bilayers in the transverse direction. They are also generally performed at constant pressure and temperature (i.e., in the NPT ensemble) under zero net bilayer tension.

Published results from MD simulations of asymmetric bilayers include investigations of the lateral organization of lipids within the two leaflets of asymmetric bilayers [79,80,84,85], the effects of asymmetry on dipole potential [5,21,86], cholesterol distribution [87,88], permeation [89], peptide-induced membrane deformation [39], and interleaflet coupling [79,90–93], to name a few. The properties of any simulated bilayer are subject to the underlying set of force field parameters, which determine the forces exerted on the individual atoms and thus the dynamics of the system. Existing force fields have been calibrated exclusively against data from symmetric bilayers. Thus, validation of the asymmetric simulation trajectories with experimental measurements is important to establish the reliability of *in silico* results. However, such validation currently presents two major challenges due to: (1) the dependence of membrane properties on the relative numbers of lipids in the two leaflets; and (2) the current inability to fully define the experimental asymmetric membranes to relate them to the computational models. Each of these challenges is explained in more detail below. It is worth, however, noting that neither of these problems is relevant for symmetric bilayers whose leaflets, by default, have the same numbers and types of lipids and are therefore, well defined and stress-free.

The first major hurdle in studying asymmetric bilayers with MD simulations is the decision of how many lipids to place in each leaflet during bilayer construction. On the timescale of most simulations, phospholipids cannot spontaneously flip between leaflets and remain in the leaflet in which they were initially placed. At the same time, the two bilayer leaflets have equal areas, a constraint imposed by the periodic boundary conditions. Thus, in simulations of flat bilayer patches where the system size normally suppresses bending deformations, it has been shown that bilayers can be stable even in the presence of a large mismatch between the numbers of lipids in the two leaflets [94–96]. This stability, however, comes at the cost of stretching and compression deformations in the under- and over-populated leaflets, respectively, potentially leading to large differences in their physical properties [72,94,95,97]. Although these effects are easy to identify in a bilayer that is composed of a single type of lipid, the contributions of interleaflet number mismatch to the resulting properties of a multi-component membrane become difficult to define. Different types of lipids have different structures (shapes), melting temperatures, and spontaneous curvatures, which determine their optimal packing in a mixture. When asymmetrically distributed across the two bilayer leaflets, the lipids may change their optimal packing preferences, due to the additional effects of interleaflet coupling. Thus, the reference state for defining the sub-optimal packing of lipids due to expansion and compression is usually not known a priori; although it could be identified with additional simulations and analysis [97].

**Figure 11.**
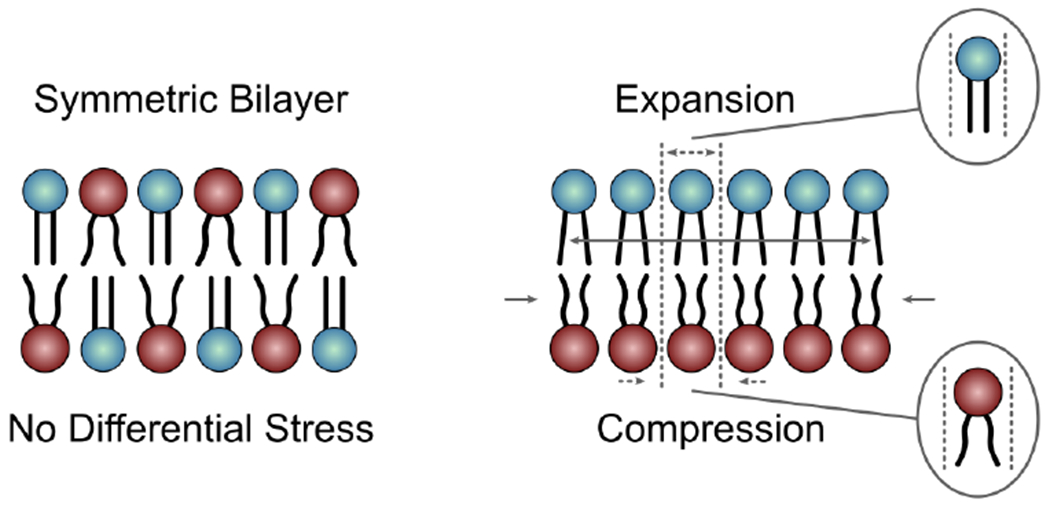
Differential stress in lipid bilayers. **Left**, a symmetric bilayer composed of two types of lipids with tensionless leaflets. **Right**, an asymmetric bilayer under differential stress, i.e., with non-zero leaflet tension. The areas of the lipids in the top and bottom leaflets are expanded and compressed, respectively, relative to their areas in the absence of leaflet tension. Adapted from [24].

One parameter that has emerged as a useful measure of stretching and compression deformations in asymmetric bilayers is leaflet tension. Negative leaflet tension indicates that the leaflet is compressed, whereas positive leaflet tension means that the lipids are laterally stretched. This parameter can be calculated from simulation trajectories by integrating the lateral pressure profile of each leaflet [98]. Leaflet tension is zero both in symmetric bilayers and in asymmetric bilayers with appropriately balanced numbers of lipids in the two leaflets (Figure 11 Left) [97]. A non-zero leaflet tension, also termed differential stress, can change the physical properties of the bilayer, including its structure and elastic constants, in a magnitude-dependent manner (Figure 11 Right) [72,97]. Therefore, in addition to the individual leaflet lipid compositions, the amount of differential stress must be taken into account when comparing different asymmetric bilayers to one another or to symmetric bilayers [97]. However, while both parameters are accessible in simulated membranes, only the leaflet lipid compositions can be determined *in vitro* [13,36]. Thus, currently, the experimental systems are not well defined, which precludes any direct comparisons with, and validation of, the corresponding computational models.

The concept of differential stress was introduced only recently as an important parameter relevant for both simulated systems and experimental model membranes [72,99,100]. Indeed, most of the simulation studies on membrane asymmetry published to date do not report on, or account for, the differential stress in the bilayers. Even if they did, it is not clear what leaflet tension would be most relevant for comparison to experimental systems. There is evidence that a non-zero leaflet tension could explain the surprising increase in bending rigidity observed in asymmetric vesicles prepared with the phase transfer technique [67]. A similar asymmetry-induced increase in the bending modulus was also reported in vesicles prepared using cyclodextrin-mediated lipid exchange [101]. These observations suggest that asymmetric model membranes *in vitro* likely harbor differential stress irrespective of how they are prepared. Its magnitude would depend on both lipid composition and the experimental conditions, but cannot be directly measured. At the same time, various parameters, and their relation to leaflet tension, can be examined with MD simulations and linked back to the *in vitro* systems via comparisons with other types of experimental data (e.g., bilayer structural parameters). Thus, moving forward a synergy between simulations and experiments could enable the estimation of leaflet tension in the experimentally prepared membranes and make possible the robust analysis, both *in vitro* and *in silico,* of fully defined asymmetric bilayer models.

## 4. Concluding Remarks

Over the last ~20 years, the field of membrane biophysics related to PM asymmetry research has expanded greatly. Although the foundational research showing that the PM of mammalian cells is asymmetric in the distribution of its chemically distinct lipids was carried out nearly 50 years ago, it had been technically challenging to produce stable asymmetric vesicles suitable for biophysical studies. This largely changed with the development of CD-mediated lipid exchange that has allowed for the reproducible preparation of stable asymmetric vesicles and has led to the development of other methods of preparing asymmetric membranes. The techniques described in this review are sufficiently developed to be used in studies that will expand our understanding of PM asymmetry, and begin to tease out the many mysteries associated with this important membrane feature.

As more research groups can prepare and study asymmetric membranes, many questions related to PM asymmetry are being addressed and undoubtedly, more will be clarified in the future. With the use of MD simulations to study PM asymmetry, a clear concern has arisen related to leaflet tension. Looking forward, it is imperative that both MD simulations and experimental techniques find ways to account for differential stress. Regarding sample preparation, a promising technique is hemifusion to produce aGUVs [32]. This technique has already demonstrated its utility by showing that stable asymmetric GUVs can be produced [32]. As lipid exchange is driven by diffusion through the hemifusion of two different membranes, potential contamination caused by residual oil or cyclodextrins are no longer a concern. There is also great promise that this technique may be able to address an important question in membrane biology, how membrane asymmetry and lipid rafts synergistically control critical membrane-based processes, such as protein signaling across cellular plasma membranes [24,102,103].
